# Novel surgical procedure for preventing anastomotic leakage following colorectal cancer surgery: A propensity score matching study

**DOI:** 10.3389/fonc.2022.1023529

**Published:** 2022-11-10

**Authors:** Gang Tang, Feng Pi, Da-Hong Zhang, Yu-Hao Qiu, Zheng-Qiang Wei

**Affiliations:** Department of Gastrointestinal Surgery, The First Affiliated Hospital of Chongqing Medical University, Chongqing, China

**Keywords:** anastomotic leakage, colorectal cancer, mesentery, perfusion, propensity score, sigmoid colon

## Abstract

Hypoperfusion is the main cause of anastomotic leakage (AL) following colorectal surgery. The conventional method for evaluating anastomotic perfusion is to observe color change and active bleeding of the resection margin of the intestine and the pulsation of mesenteric vessels. However, the accuracy of this method is low, which may be due to insufficient observation time. A novel surgical procedure that separates the mesentery in advance at the intended transection site can delay the observation of anastomotic perfusion, and can potentially detect more anastomotic sites with insufficient vascular supply and reduce the rate of AL. This study aimed to investigate the effects of a novel surgical procedure on AL following sigmoid colon and rectal cancer surgeries. A total of 343 patients who underwent rectal and sigmoid colon cancer surgeries were included in the study. From August 2021 to June 2022, patients with sigmoid colon or rectal cancer underwent a new surgical procedure of pre-division of the mesentery (PDM) at the intended transection site (PDM group). Patients with colorectal cancer who underwent conventional surgical procedures from August 2018 to July 2021 were categorized as the non-PDM group. Symptomatic AL (SAL) within 30 days and other outcomes were retrospectively analyzed using propensity score matching and compared between the two groups. The incidences of SAL were 1.3% and 11.3% in the PDM and non-PDM groups, respectively. PDM significantly reduced the SAL rate in sigmoid colon and rectal cancer surgeries (P = 0.009). The incidence of total postoperative complications (P < 0.05) was significantly lower in the PDM group than that in the non-PDM group. There were no significant differences between the two groups for operative time (P = 0.662), intraoperative blood loss (P = 0.651), intraoperative blood transfusion (P = 0.316), and intensive care rate (P = 1). The length of postoperative hospital stay (P = 0.010) and first exhaust (P = 0.001) and defecation time (P < 0.05) were shorter in the PDM group than in the non-PDM group. PDM can effectively prevent AL, and this procedure can be safely performed in sigmoid colon and rectal cancer surgeries.

## Introduction

Anastomotic leakage (AL) is one of the major postoperative complications of colorectal surgery, and is the main cause of increased postoperative morbidity and mortality (1, 2). AL not only leads to increased medical costs and longer hospital stays, but also affects patients’ outcomes (3, 4). A meta-analysis showed that the occurrence of AL increases the rate of local recurrence and reduces long-term patient survival (5). In the past few decades, with the advancement of medical technology, postoperative complications of colorectal cancer surgery have decreased, but AL still poses a problem for colorectal surgeons (6, 7).

Risk factors for AL include male sex, obesity, low tumor location, large tumor diameter, advanced tumor stage, preoperative hypoproteinemia, neoadjuvant chemoradiotherapy, smoking, diabetes, poor nutritional status, and poor blood perfusion in the anastomotic area. Of these factors, anastomotic blood perfusion influences AL the most (6, 8, 9); therefore, the detection of ischemic anastomotic tissue is key in preventing AL during surgery. The conventional method for evaluating anastomotic perfusion is to observe color change and active bleeding of the resection margin of the intestine and the pulsation of mesenteric vessels; however, the accuracy of conventional evaluation strategies are low (7), and may be due to insufficient observation time. When blood perfusion of the anastomotic tissue is slightly poor, color changes may not be observed in a short period of time. Therefore, to improve the accuracy of conventional surgical procedures, we proposed to extend the observation time to detect more anastomotic sites that display poor blood perfusion. We proposed to separate the mesenteric membrane of the intended transection site after ligating the inferior mesenteric artery, which is followed by the complete dissociation of the colon and rectum. Then, the rectum or colon at the distal end of the tumor is dissected, and perfusion can be assessed at the intended transection site.

No clinical studies have yet been conducted to confirm the effectiveness of this new surgical procedure in preventing AL. Propensity score matching (PSM) is a statistical method that reduces the imbalances in baseline data between experimental and control groups (10). Furthermore, the incidences of AL following surgeries on the sigmoid colon and rectum were higher than that of the right colon (11). Therefore, we used PSM to explore the effects of the new surgical procedure on postoperative AL in patients with sigmoid colon and rectal cancers.

## Methods

### Study population

This study was approved by Ethics Committee of The First Affiliated Hospital of Chongqing Medical University (Approval No. 2022-K343) and all patients signed informed consents. We retrospectively analyzed 343 consecutive patients who underwent surgery for rectal and sigmoid colon cancers in the Department of Gastrointestinal Surgery, The First Affiliated Hospital of Chongqing Medical University from August 2018 to June 2022.

We included patients with pathologically confirmed sigmoid colon or rectal cancer undergoing elective laparoscopic or robot-assisted laparoscopic surgery. The exclusion criteria were as follows (1): cases without primary anastomosis (2), cases with multi-visceral resections (3), patients undergoing laparoscopic surgery that were converted to open surgery (4), open surgery cases (5), patients with a history of treatment for other abdominal or pelvic malignancy (6), multiple primary cancers, and (7) emergency cases.

Between August 2021 and June 2022, all patients with sigmoid colon or rectal cancer underwent a new surgical procedure of pre-division of the mesentery (PDM) at the intended transection site to delay the observation of tissue perfusion at the intended transection site. This group of patients was categorized as the PDM group. Patients with colorectal cancer who underwent conventional surgical procedures from August 2018 to July 2021 were categorized as the control or non-PDM group.

### Surgical procedure

**Figure 1:**
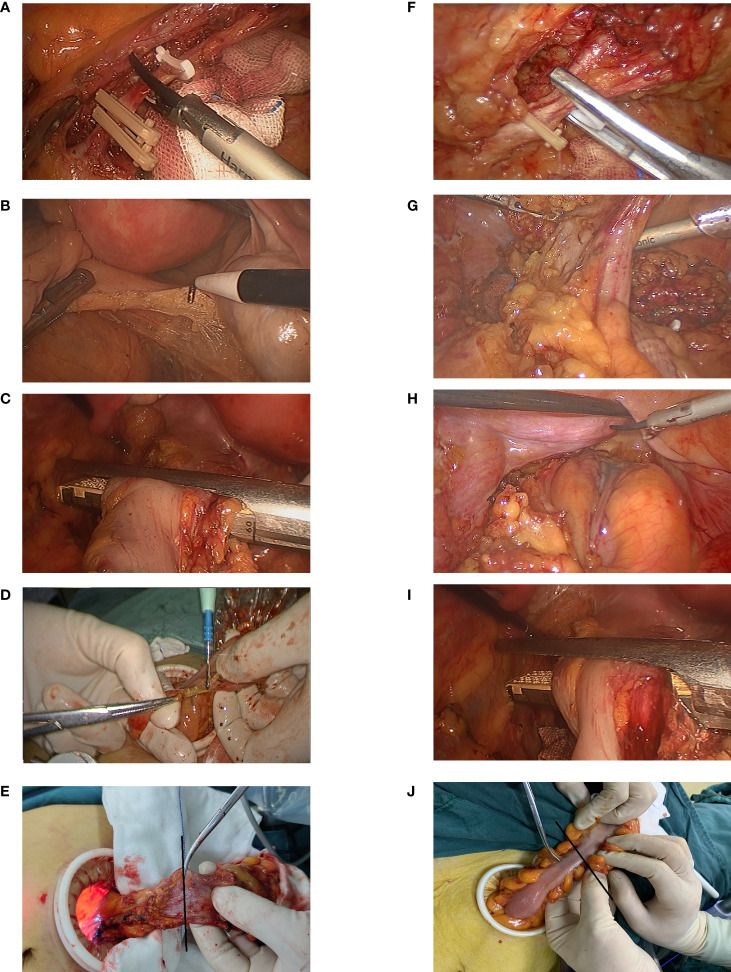
Surgical procedure. In the non-PDM group, surgical procedures included **(A)** ligation of the inferior mesenteric artery proximal to its origin. **(B)** After separating the distal rectum, **(C)** the anal side of the rectum was transected and the specimen was extracted through the abdominal incision. After **(D)** dissection of the mesentery at the level of planned transection (black line), **(E)** Vascular perfusion was observed. In the PDM group, **(F)** the inferior mesenteric artery was ligated proximal to its origin. **(G)** the mesentery of the intended transection site with a length greater than 3 cm was separated. After **(H)** separating the distal rectum, **(I)** the anal side of the rectum was transected. **(J)** The vascular perfusion was observed at the level of planned transection (black line) after the specimen was removed from the abdominal incision.

Our hospital has adopted standardized laparoscopic colorectal cancer and robotic surgeries, which were performed by two experienced surgeons. Poke cards were inserted around the umbilicus to establish a pneumoperitoneum at a pressure of 10 mmHg, and then 4 additional poke cards were inserted. High ligation of the inferior mesenteric artery was performed in all patients. The decision to reduce the spleen flexion depended on intraoperative intestinal tension. Intraperitoneal or pelvic drainage tubes were routinely placed during the surgery. For the distal rectum, a linear stapler under laparoscopy was used to make a transverse incision *in vivo*. After tumor specimens were resected, the proximal sigmoid colon and rectal stump were anastomosed using an end-to-end double stapler technique. Standard air leak tests were routinely performed, and the tissue rings were checked for integrity. When there was leakage of air or the anastomotic effect was not good, suturing was performed to reinforce the anastomosis. In the PDM group, the new procedure of delayed observation was performed; after ligation and dissection of the inferior mesenteric artery and dissociation of the proximal colon, the mesentery of the intended transection site with a length greater than 3 cm was separated, followed by the dissociation of the distal colon or rectum. The distal rectum was transected and the specimen was removed from the abdominal incision. Perfusion of the intended transection site of the bowel was observed prior to proximal resection. The time between the separation of the mesentery at the intended transection site and the beginning of observation was defined as delayed observation time. In the non-PDM group, conventional surgical procedures were performed; after ligation and dissection of the inferior mesenteric artery, sufficient length of colon or rectum was dissociated. Then, the distal rectum was transected, and the tissue perfusion at the planned transection level was observed immediately after the mesentery at the planned transection site and was separated (**Figure 1**).

### Primary and secondary endpoints

The primary endpoint of our study was the incidence of symptomatic AL within 30 days after surgery. Secondary endpoints were blood loss, operative time, intraoperative blood transfusion rate, incidence of postoperative complications, time to first exhaust and defecation, length of hospital stay, rate of surgical plan change, rate of reoperation within 30 days after surgery, and mortality. Demographic and perioperative data of the patients were retrieved from electronic medical records.

### Definition of anastomotic leakage

The definition of AL outlined by the International Study Group for Rectal Cancer was used in this study. AL was classified into three grades. Grade A is known as asymptomatic AL, where no clinical symptoms or laboratory abnormalities is detected from the leakage. Grade B and C are referred to as symptomatic AL (SAL). Grade B requires aggressive therapeutic intervention but does not require surgery, while grade C AL requires reoperation (12). Only SAL was analyzed in this study, since the use of contrast enema to detect asymptomatic AL after surgery was not routinely performed.

### Statistical analysis

The sample size was estimated using the chi-square test with a significance level of 0.05 (double-sided) and power of 0.80. Previously published studies showed that the incidence of SAL in the control group was 13.7%. Based on previous findings, assuming a 2% incidence of SAL in the PDM group, we estimated that 65 cases need to be included in each matched group.

To reduce selection bias caused by potential confounding factors, PSM was performed based on the following risk factors associated with AL: gender, age, body mass index (BMI), smoking history, physical condition according to the classification system of the American Society of Anesthesiologists, preoperative neoadjuvant therapy, tumor location, the use of transanal tube, tumor stage, diverting stoma, air leak test, and surgical approach. The nearest neighbor matching method was used for one-to-one matching, and the caliper size was set to 0.05 standard deviation of the logarithm of the estimated propensity score. Pearson’s chi square test and Mann–Whitney U test were used to compare categorical and continuous variables, respectively. Statistical analyses were performed using IBM SPSS version 26. All P values were two-sided, and P < 0.05 was considered significant.

## Results

### Patients’ characteristics

A total of 343 patients were included in this study. The baseline characteristics of the included patients are shown in **Table 1**. There were 83 and 260 patients in the PDM and non-PDM groups, respectively. There were 27 and 70 cases of sigmoid colon cancer and 56 and 190 cases of rectal cancer in the PDM and non-PDM groups, respectively. The proportion of rectal cancer in the two groups was not significantly different (P = 0.323). Before PSM analysis, preoperative serum albumin level (P = 0.000) and clinical stage (P = 0.017) were significantly different between the two groups. There were 3 and 13 cases with positive air leakage test in the PDM and non-PDM groups, respectively, and all of them underwent anastomotic reinforcement. One case with positive air leak test in the non-PDM group underwent protective stoma. In only one case in the non-PDM group the tissue ring was found to be incomplete after the anastomosis and anastomotic reinforcement and protective stoma were performed. The incidence of SAL (**Table 2**) was significantly lower in the PDM group than in the non-PDM group (1.2% vs. 8.5%; P = 0.021). In addition, the incidences of grade B and C AL (**Table 3**) were significantly higher in the PDM (1.2% and 0%, respectively) than in the non-PDM group (6.2% and 2.3%, respectively).

**Table 1:** Baseline characteristics and tumor locations before propensity score matching.

	Group PDM (n = 83)	Group Non-PDM (n = 260)	P value
Age (years)^a^	65 (57-74)	62.5 (54-71)	0.085
Gender (%)			0.962
Male	51 (61.4)	159 (61.2)	
Female	32 (38.6)	101 (38.8)	
BMI^a^	23.5 (21.7-25.1)	23.3 (21.4-25.7)	0.660
Albumin (g/dL)^a^	3.9 (3.6-4.1)	4.1 (3.8-4.4)	0.000
Smoking within a year (%)	22 (26.5)	67 (25.8)	0.894
Preoperative Ileus (%)	20 (24.1)	55 (21.2)	0.572
Diabetes Mellitus (%)	13 (15.7)	39 (15.0)	0.883
Coronary artery disease (%)	9 (10.8)	17 (6.5)	0.197
ASA Grade (%)			0.478
1	3 (3.6)	4 (1.5)	
2	48 (57.8)	148 (56.9)	
3	32 (38.6)	108 (41.5)	
Neoadjuvant therapy received (%)	8 (9.6)	26 (10.0)	0.924
Treatment modality (%)			0.144
Robotic	24 (28.9)	55 (21.2)	
Laparoscopy	59 (71.1)	205 (78.8)	
Distance between tumor and AV (cm)^a^	10 (7-20)	10 (6-16)	0.235
Positive air leak test	3 (3.6)	13 (5)	0.602
Diverting stoma (%)	14 (16.9)	48 (18.5)	0.742
Transanal tube (%)	25 (30.1)	79 (30.4)	0.964
Stapled Functional end to end anastomosis (%)	83 (100.0)	260 (100.0)	
UICC stage (%)			0.017
I	17 (20.5)	41 (15.8)	
II	39 (47.0)	92 (35.4)	
III	17 (20.5)	103 (39.6)	
IV	10 (12.0)	24 (9.2)	

Values in parentheses are percentages, unless indicated otherwise; ^a^ values are median (interquartile range: 25-75th percentile).

ASA, American Society of Anaesthesiologists physical status classification; AV, anal verge; BMI, Body mass index; PDM, pre-division of the mesentery; UICC, Union for International Cancer Control.

**Table 2:** Operative outcomes and postoperative complication before propensity score matching.

	Group PDM (n=83)	Group Non-PDM (n=260)	P value
Duration of surgery (min)^a^	224 (165-263)	200 (170-245)	0.216
Intraoperative blood loss (ml)^a^	50 (30-60)	50 (30-50)	0.751
The time from separation of the planned crosscutting level of the mesentery to evaluation of the planned crosscutting level (min)^a^	49 (43-62)		
Change in surgical plan (%)	9 (10.8)		
Transfusion (%)	0 (0)	3 (1.2)	0.326
Reoperation (%)	0 (0)	7 (2.7)	0.131
Mortality (%)	0 (0)	0 (0)	
Intensive care (%)	3 (3.6)	8 (3.1)	0.809
Time of first exhaust (days)^a^	1 (1-2)	2 (1-2)	0.165
The time of first bowel movement (days)^a^	2 (2-3)	3.5 (3-4)	0.000
Hospital stay (days)^a^	8 (7-10)	8 (7-10)	0.063
Postoperative complications (%)	29 (34.9)	127 (48.8)	0.027
Complications not related to anastomosis (%)	27 (32.5)	101 (38.8)	0.300
Urinary infection (%)	1 (1.2)	3 (1.2)	0.970
Pneumonia (%)	1 (1.2)	14 (5.4)	0.105
Ileus (%)	1 (1.2)	13 (5.0)	0.128
Wound infection (%)	1 (1.2)	6 (2.3)	0.536
Intraabdominal infection (%)	18 (21.7)	57 (21.9)	0.964
Anastomotic leakage (%)	1 (1.2)	22 (8.5)	0.021
Bleeding at anastomotic site (%)	1 (1.2)	4 (1.5)	0.825
Others (%)	5 (6.0)	8 (3.1)	0.221

Values in parentheses are percentages, unless indicated otherwise; ^a^ values are median (interquartile range: 25-75th percentile). PDM, pre-division of the mesentery.

**Table 3:** Postoperative Anastomotic leakage.

Anastomotic leakage	Group PDM (before PSM n = 83)	Group Non-PDM (before PSM n = 260)	P value	Group PDM (after PSM n = 80)	Group Non-PCM (after PSM n = 80)	P value
Symptomatic Anastomotic leakage (%)	1 (1.2)	22 (8.5)	0.021	1 (1.3)	9 (11.3)	0.009
Grade B (%)	1 (1.2)	16 (6.2)	0.071	1 (1.3)	7 (8.8)	0.030
Grade C (%)	0 (0)	6 (2.3)	0.163	0 (0)	2 (2.5)	0.155

Values in parentheses are percentages. PSM, propensity score matching.

### Propensity score matching analysis

Due to the heterogeneity of covariables between the PDM and non-PDM groups, PSM was performed. After matching (n =160), there were no significant differences between the PDM (n=80) and non-PDM groups (n=80) for all covariables (i.e., age, gender, BMI, preoperative serum albumin level, smoking within a year, preoperative ileus, diabetes mellitus, American Society of Anesthesiologists physical condition score, coronary artery disease, neoadjuvant therapy, treatment modality, distance between tumor and anal verge, diverting stoma, air leak test, and transanal tube) (**Table 4**). All patients had intact tissue rings. The median time from the separation of the intended transection level of the mesentery to evaluation of the planned crosscutting level was 49.5 minutes (interquartile range: 43–63.5 minutes). In the PDM group, eight patients (10%) had poor perfusion, and the planned transection point was subsequently changed.

**Table 4:** Baseline characteristics and tumor locations after propensity score matching.

	Group PDM (n = 80)	Group Non-PDM (n = 80)	P value
Age (years)^a^	65 (57-73.8)	63 (55-71)	0.461
Gender (%)			0.872
Male	48 (60.0)	47 (58.8)	
Female	32 (40.0)	33 (41.3)	
BMI^a^	23.5 (21.7-25.2)	23.0 (21.0-25.9)	0.507
Albumin (g/dL)^a^	3.9 (3.7-4.1)	4.0 (3.7-4.3)	0.250
Smoking within a year (%)	21 (26.3)	18 (22.5)	0.581
Preoperative Ileus (%)	18 (22.5)	16 (20.0)	0.699
Diabetes Mellitus (%)	12 (15.0)	13 (16.3)	0.828
Coronary artery disease (%)	8 (10.0)	8 (10.0)	1
ASA Grade (%)			0.755
1	2 (2.5)	3 (3.8)	
2	46 (57.5)	49 (61.3)	
3	32 (40.0)	28 (35.0)	
Neoadjuvant therapy received (%)	8 (10.0)	7 (8.8)	0.786
Treatment modality (%)			1.000
Robotic	23 (28.8)	23 (28.8)	
Laparoscopy	57 (71.3)	57 (71.3)	
Distance between tumor and AV (cm)^a^	10 (6.3-18)	10 (5.1-18)	0.605
Positive air leak test	3 (3.8)	4 (5.0)	0.699
Diverting stoma (%)	14 (17.5)	10 (12.5)	0.376
Transanal tube (%)	24 (30.0)	34 (42.5)	0.100
Stapled Functional end to end anastomosis (%)	80 (100.0)	80 (100.0)	
UICC stage (%)			0.285
I	17 (21.3)	12 (15.0)	
II	37 (46.3)	35 (43.8)	
III	17 (21.3)	27 (33.8)	
IV	9 (11.3)	6 (7.5)	

Values in parentheses are percentages, unless indicated otherwise; ^a^ values are median (interquartile range: 25-75th percentile).

ASA, American Society of Anaesthesiologists physical status classification; AV, anal verge; BMI, Body mass index; PDM, pre-division of the mesentery; UICC, Union for International Cancer Control.

The incidences of SAL (i.e., grade B and C) were 1.3% (1/80) and 11.3% (9/80) in the PDM and non-PDM groups (P = 0.009), respectively (**Table 3**). PDM significantly reduced the SAL rate in sigmoid colon and rectal cancer surgeries. The incidence of grade B AL in the PDM group was significantly lower than that in the non-PDM group (p = 0.030). The incidences of reoperation due to AL (i.e., grade C) were 0% (0/80) and 2.5% (2/80) in the PDM and non-PDM groups, respectively. The rates of total postoperative complications was significantly lower in the PDM group (32.5%) than in the non-PDM group (72.5%) (**Table 5**). In addition, there were no significant differences between the two groups for operative time (P = 0.662), intraoperative blood loss (P = 0.651), intraoperative blood transfusion (P = 0.316), and intensive care rate (P = 1). PDM reduces the time to first exhaust (P = 0.001) and defecation (P < 0.05). The length of postoperative hospital stay was shorter (P = 0.010) in the PDM group than in the non-PDM group. No deaths were reported in either group.

**Table 5:** Operative Outcomes and postoperative complication after propensity score matching.

	Group PDM (n=80)	Group Non-PDM (n=80)	P value
Duration of surgery (min)^a^	222 (164.3-263)	212.5 (181-243.8)	0.662
Intraoperative blood loss (ml)^a^	50 (30-50)	50 (30-57.5)	0.651
The time from separation of the planned crosscutting level of the mesentery to evaluation of the planned crosscutting level (min)^a^	49.5 (43-63.5)		
Change in surgical plan (%)	8 (10.0)		
Transfusion (%)	0 (0)	1 (1.3)	0.316
Reoperation (%)	0 (0)	3 (3.8)	0.080
Mortality (%)	0 (0)	0 (0)	
Intensive care (%)	2 (2.5)	2 (2.5)	1.000
Time of first exhaust (days)^a^	1 (1-2)	2 (1-2.8)	0.001
The time of first bowel movement (days)^a^	2 (2-3)	4 (3-5)	0.000
Hospital stay (days)^a^	8 (7-9.8)	8.5 (7-11)	0.010
Postoperative complications (%)	26 (32.5)	58 (72.5)	0.000
Complications not related to anastomosis (%)	24 (30.0)	47 (58.8)	0.000
Urinary infection (%)	1 (1.3)	1 (1.3)	1.000
Pneumonia (%)	1 (1.3)	7 (8.8)	0.030
Ileus (%)	1 (1.3)	8 (10.0)	0.016
Wound infection (%)	1 (1.3)	4 (5.0)	0.173
Intraabdominal infection (%)	15 (18.8)	25 (31.3)	0.068
Anastomotic leakage (%)	1 (1.3)	9 (11.3)	0.009
Bleeding at anastomotic site (%)	1 (1.3)	2 (2.5)	0.560
Others (%)	5 (6.3)	2 (2.5)	0.246

Values in parentheses are percentages, unless indicated otherwise; ^a^ values are median (interquartile range: 25-75th percentile). PDM: pre-division of the mesentery.

Among patients with rectal cancer, the incidence of SAL was significantly lower in the PDM group (0%) than in the non-PDM group (11.1%) before matching (P = 0.009). After matching, all variables (i.e., age, gender, BMI, preoperative serum albumin level, smoking within a year, preoperative ileus, diabetes mellitus, American Society of Anaesthesiologists physical condition score, coronary artery disease, neoadjuvant therapy, treatment modality, distance between tumor and anal verge, diverting stoma, air leak test, and transanal tube) were balanced between the two groups; there were 56 rectal cancer patients in each group. The incidences of SAL were 0% (0/56) and 10.7% (6/56) in the PDM and non-PDM groups, respectively. PDM significantly reduced the incidence of SAL (P = 0.012) and the incidence of total complications (P < 0.05) after rectal cancer surgery (**Table 6**).

**Table 6:** Operative Outcomes and postoperative complication in rectal cancer.

	Group PDM (before PSM n=56)	Group Non-PDM (before PSM n=190)	P value	Group PDM (after PSM n=56)	Group Non-PCM (after PSM n=56)	P value
Duration of surgery (min)^a^	240 (176.3-287.5)	215 (180-250.5)	0.098	240 (176.3-287.5)	205 (181.3-244.3)	0.155
Intraoperative blood loss (ml)^a^	50 (30-90)	50 (30-62.5)	0.955	50 (30-90)	50 (30-50)	0.886
Hospital stay (days)^a^	8 (7-9.8)	8 (7-11)	0.033	8 (7-9.8)	9 (7-11.8)	0.047
Postoperative complications (%)	13 (23.2)	104 (54.7)	0.000	13 (23.2)	36 (64.3)	0.000
Anastomotic leakage (%)	0 (0)	21 (11.1)	0.009	0 (0)	6 (10.7)	0.012
Grade B (%)	0 (0)	15 (7.9)	0.030	0 (0)	4 (7.1)	0.042
Grade C (%)	0 (0)	6 (3.2)	0.178	0 (0)	2 (3.6)	0.154

Values in parentheses are percentages, unless indicated otherwise; ^a^ values are median (interquartile range: 25-75th percentile). PDM: pre-division of the mesentery. PSM: propensity score matching.

## Discussion

Our study showed that PDM can effectively reduce the postoperative incidence of SAL following sigmoid colon and rectal cancer surgeries. In addition, compared with the control group, PDM did not increase the operative time, amount of intraoperative blood loss, or transfusion rate. Furthermore, when PDM was performed, the incidence of total postoperative complications and length of hospital stay were shortened, suggesting that PDM is a safe and feasible surgical procedure in preventing AL in sigmoid and rectal cancer surgeries.

The estimated incidence of AL after colon surgery is between 6–8%, while it is as high as 7–20% for rectal surgery (13). AL is associated with increased postoperative morbidity, mortality, length of hospital stay, recurrence of cancer, permanent stoma, and total costs (3, 14, 15). Capolupo et al. (16) showed that the cost and length of hospital stay of patients with AL was approximately twice of that of patients without AL.

To prevent AL, several strategies have been proposed, but the effectiveness of most of them are still controversial. For example, indocyanine green (ICG) fluorescence angiography may be a potential strategy to prevent AL (17). Several studies have shown that intraoperative ICG fluorescence angiography can effectively reduce the incidence of AL (3, 7, 18). However, two recent large randomized controlled trials (RCTs) (19, 20) showed no significant difference in the incidence of AL between the ICG and control groups. In addition, intraoperative ICG fluorescence angiography requires special endoscopy instruments, and only a few hospitals are equipped with fluorescence endoscopy instruments. It may be difficult to conduct ICG fluorescence angiography, especially in poor areas. Protective stoma is a conventional strategy to prevent AL, but its efficacy remains controversial. A meta-analysis showed that protective stoma can reduce the incidence of AL, but the evidence is weak, and routine preventive stoma for rectal cancer requires high-quality evidence (21), and stoma-related complications can occur (21, 22). Transanal decompression tubes are also widely used to prevent AL. Some retrospective studies (23, 24) have shown that the use of transanal drainage tubes can effectively reduce the rate of AL; however, two recent RCTs (25, 26) showed that the use of transanal tube did not reduce AL.

The risk factors of AL in this study were male sex, obesity, low tumor location, large tumor diameter, advanced tumor, preoperative hypoproteinemia, neoadjuvant chemoradiotherapy, smoking, diabetes, poor nutritional status, and poor blood perfusion in the anastomotic area (6, 8, 9). Due to the difficulty in controlling risk factors such as age, sex, tumor location, comorbidities, tumor stage and nutritional status, surgeons have to focus on anastomotic techniques, anastomotic tension, and anastomotic blood perfusion (27). Anastomotic hypoperfusion is a recognized cause of AL and stenosis (28, 29). However, traditional assessment strategies are unreliable and have low specificity (28). This may be related to the insufficient observation time of anastomotic tissue perfusion. In conventional surgical procedures, the anal side of the rectum is excised, and specimens are removed through an umbilical incision. Tissue perfusion is evaluated after mesentery separation at the transverse level prior to proximal bowel resection. Due to the short observation period, some areas of the intestine at the intended transection level with slight ischemia are not detected because it does not display any difference from the area of intestine with normal blood perfusion, which may lead to the occurrence of postoperative AL. In our study, the median time between the separation of the mesangium and observation of tissue perfusion was 49.5 minutes, and the longer time interval made it easier to identify the color changes of the intestine with tissue ischemia and active bleeding at the cutting edge. This was further confirmed by our results, in which eight cases (8/80) with poor tissue blood supply were identified in the PDM group and the intended transection line was changed. We can assume that, without the change in crosscutting due to PDM, the leakage rate would have been 11.3% in the PDM group and 11.3% in the entire cohort, and these results are consistent with the estimated incidence in previous studies. Since patients with rectal cancer are more likely to develop AL after surgery, we analyzed these patients separately and found that PDM can effectively reduce the incidence of AL. In addition, in our study, the length of intestinal function recovery time and hospital stay of the PDM group were shorter than those of the control group, which may be related to the lower incidence of AL in the PDM group. Studies have also shown that AL is associated with an increase in short-term complications (4), which aligns to the findings in our study. Moreover, the non-PDM group had a higher incidence of AL and total postoperative complications, such as pneumonia and intestinal obstruction, than the PDM group. Although no significant difference was observed in the rate of reoperation due to AL between the two groups, this may be due to the small sample size of our study. Prospective, large sample studies are needed to confirm the effect of PDM on reoperation due to AL.

Our novel surgical procedure has two advantages. First, no additional equipment or technical support is required; thus, our procedure does not impose any additional economic burden and is easy to promote. Second, it is simple and relatively easy to learn, without the addition of complex surgery steps. However, one drawback of our procedure is that it requires the surgeon to separate the mesentery in a predetermined cross section under a laparoscope, which may be difficult for inexperienced surgeons.

There are some limitations to our study. First, this study was a single-center retrospective study. Second, the design of this study was non-random, and it was impossible to control for every deviation in the study. Therefore, PSM was used to reduce the deviations between groups. Third, grade A AL had little impact on the prognosis of patients with colorectal cancer, and no intervention was required. Contrast agent enema examination was not routinely performed; thus, our study did not evaluate the effect of PDM on grade A AL.

In conclusion, our study indicated that PDM can effectively prevent AL, and can be safely performed in sigmoid colon and rectal cancer surgeries. Although the results of our study need to be further confirmed by RCTs, our study provided a reference for the development of a potential strategy for preventing AL.

## Data availability statement

The raw data supporting the conclusions of this article will be made available by the authors, without undue reservation.

## Ethics statement

This study was approved by Ethics Committee of The First Affiliated Hospital of Chongqing Medical University (Approval No. 2022-K343). The patients/participants provided their written informed consent to participate in this study.

## Author contributions

Conceptualization: Z-QW, FP, GT, and D-HZ. Data collection and analyses: FP, GT, D-HZ, and Y-HQ. Writing—original draft preparation: GT and FP. Writing—review and editing: Z-QW, FP, GT, D-HZ, and Y-HQ. Z-QW, FP, GT, D-HZ, and Y-HQ had primary responsibility for final content. All authors contributed to the article and approved the submitted version.

## Funding

This study was funded by Chongqing Key Diseases Research and Application Demonstration Program, No. 2019ZX003.

## Conflict of interest

The authors declare that the research was conducted in the absence of any commercial or financial relationships that could be construed as a potential conflict of interest.

## Publisher’s note

All claims expressed in this article are solely those of the authors and do not necessarily represent those of their affiliated organizations, or those of the publisher, the editors and the reviewers. Any product that may be evaluated in this article, or claim that may be made by its manufacturer, is not guaranteed or endorsed by the publisher.
